# Brainard’s Case of Ununited Fracture of the Femur

**Published:** 1848

**Authors:** Daniel Brainard

**Affiliations:** Professor of Surgery in Rush Medical College


THE NORTH-WESTERN

### MEDICAL & SURGICAL JOURNAL.

Vol. I.] AUGUST AND SEPTEMBER, 1848. [No. 3.

### Part 1.—Original Communications.

### ARTICLE I.

*Case of Ununited Fracture of the Femur of one year's standing, successfully treated by Resection, Denudation, and Retaining the ends of Bone by means of Wire.* By Daniel Brainard, M.D., Professor of Surgery in Rush Medical College.

About the middle of February, 1848, Sewell Rice, of Allegan county, Michigan, aged 34 years, laborer, of pretty good health and constitution, applied to me on account of an ununited fracture of the left femur.

*State of the Parts.*—On examination, the fracture was found to be situated seven inches above the knee, very oblique, the superior fragment placed in front, the ends overlapping, when no extension was made, not less than four inches, and the fragments so moveable that they seemed to have no connection with each other or with the surrounding parts. The extremity of the upper fragment in particular, when acted on by the muscles, and drawn inwards, forwards, or outwards, could be felt as if beneath the integuments only, and seemed to move as if in a sac. Both ends could be felt rounded; there was no tenderness and the member could be bent almost to a right angle at the point of fracture, without giving pain. The member hung dangling, entirely useless; the thigh, from the want of use and the pressure of an apparatus which he kept buckled around it, was atrophied and attenuated to a remarkable degree, while the foot and leg, from the obstructed circulation, were swollen, œdematous, and indurated, so that it but partially regained its natural appearance when the pressure was removed, and the patient placed in a horizontal position.

*History.*—On the 15th of March, 1847, a tree fell upon him, and not only fractured the thigh, but severely bruised the knee, leg, and ankle of the same side. There was much bruise at the point of fracture, but the skin was not broken.

The limb was laid upon a double inclined plain, the leg enveloped in a roller, and a piece of flannel pinned about the thigh. Extension was made by means of "half a brick" suspended from a cord attached to the foot, and passing over the foot of the bed, and counter extension by means of a "flat-iron" suspended from a cord attached to the pelvis and passing over the head of the bed. At the end of five and a half weeks, the weight attached to the foot was increased to about sixteen pounds, and the limb was placed in a different apparatus. During the whole of the treatment the roller upon the leg was removed every three or four days, and the member at such times subjected to considerable movement. At the end of eleven weeks, the apparatus was taken off, and the patient removed about a mile, and directed to rise and walk on crutches. This, however, he was unable to do. After sometime the surgeon examined it and finding it ununited, moved the fragments freely, and re-applied the apparatus for seven weeks.

During the whole of this time the point of fracture remained very tender; and during the night or when it was moved, there was spasmodic action of the muscles. which drew forward the upper fragment, "so that it seemed as if it would come through the skin," and shortened the member very much. Such was the history as obtained from the patient himself.

*Treatment.*—This case presented nearly every unfavorable circumstance for successful treatment which could be met with in a patient of tolerably good health and constitution. Hence, in selecting the plan of treatment, it was necessary to look at once to those of a severer character.

Thus, rest and immobility had been tried to a certain degree, but what could be expected from this when, far from being able to bring the ends of the bones together, their sides could scarcely be so approximated as not to have soft tissues intervening between them. Pressure, rubbing the bones together, cutting down and applying caustic to their ends were all evidently insignificant in this case. The seaton, regarded in this country as *the remedy* for ununited fractures is hopeless in cases of this kind; and it is from its having been used indiscriminately in these and others of a similar nature, that it has lost, in foreign countries, the credit to which it is entitld. Resection was the only means offering some chances of success, but when we consider that the want of union was due, in all probability, to the spasmodic action of the muscles, there was evidently no security against the recurrence of the same cause after the operation. In order to guard against this result, I determined to put in practice a means which, as far as I have been able to learn, has not been used in similar cases. This method consists in cutting down to the bone, denuding or removing the ends, and then passing a wire around the extremities slightly overlapping each other, and twisting the ends of the wire together so as to bind the fragments immovably in this position.

I am aware that the wire has been recommended in cases of fracture of the jaw, being carried round the teeth, in cases of fractures of the petella and in cases of compound fractures; but all these cases are widely different from the present. In case of fracture of the jaw, its application to the teeth is always harmless and often useful. In case of simple fracture of the petella, which, with good treatment, unites to form a useful member, cutting down to perforate the fragments, and hold them together with wire, is a useless and most dangerous operation, converting, as it does, a simple into a compound fracture, with opening into the articulation of the knee. But the principles applicable to that location are not to be generalized. It is obvious that in cases of ununited fractures with great mobility, the use of the wire may be made to answer the purpose of a seaton in exciting inflammation, while it holds the bones immovably in contact. In cases requiring resection or denudation it may be used as an additional means of security, or as a substitute, without increasing the danger. In many cases of superficially placed bones, its application is free from danger; and, while it holds them firmly in contact, its presence excites a moderate degree of inflammation.

Any one who will take the pains to examine the report of cases pseudar-throsis, given in different periodicals, will not fail to notice a certain number in which the use of this means is obviously indicated according to the well established principles of surgery. In some rare cases it will enable us to succeed when other means alone would fail.

These cases present themselves under such a variety of forms, situations, and circumstances, that no method of treatment is adapted to more than a small number, and new resources are still required for several varieties rebellious to the ordinary resources of art. When such cases present themselves to the surgeon, he should rather follow the indications and views which they suggest to his mind, than abandon it because the methods most approved are either inapplicable or unsuccessful. Guided by such views which we hope may be sufficient to justify us in using a means which some surgeons have, from its application in particular cases, condemned, we proceeded on the 1st of March, 1848, with the assistance of Drs. Herrick and Blaney, and in presence of the class in attendance on our spring course of lectures, to perform the operation in the following manner:

An incision was made four and a half inches in length, upon the anterior and exterior side of the thigh, and carried down to the fracture. The ends of the bones were exposed and found to be covered with a tough fibrous tissue of great firmness. This was removed by means of the bone nippers from the end of the lower fragment; the end of the upper piece being very sharp, and having great tendency to project forwards, a chain saw was passed around it, and about an inch of it removed.

Extension being then made, the two extremities were approximated to each other, and a piece of silver wire, (like that used for constricting polypi,) doubled and twisted, was slipped over each end so as to encircle the two bones. The ends of the wire were then twisted together with sufficient firmness to prevent movement without exercising much pressure on the surface of the bones. The limb was then placed in an angular splint, the sides of the wound brought together with stitches of interrupted suture and splints applied so as to restrain its movements. The operation lasted perhaps half an hour, was well borne by the patient, and no vessel required a ligature. It was evidently, so far as the immediate depressing effects were concerned, much less severe than amputation of the thigh. Using the chain saw, instead of turning out the ends of the bones to saw them off, renders the operation less severe. Considerable inflammation followed, which was combatted with anti-phlogistic treatment. The wound healed by the first intention excepting at two points of sufficient extent to allow of the escape of matter. On the third day the spasm of the muscles of the thigh, before mentioned as having followed the fracture, occurred, being excited by the slightest touch or motion. It was so severe as to shorten the limb somewhat, and was attended with such violent efforts at displacement as to show conclusively the necessity of the wire, or some equally effective restraining means. The suppuration diminished, the patient's appetite returned, and he was in every way comfortable.

The object of the wire—that of holding the ends of the bone firmly in contact until adhesions might be formed—having been accomplished, it was desirable to remove it. This was done on the 24th of March, as carefully as possible, but some inflammation followed, and on the 4th of April, fluctuation was perceived on the inside of the thigh, and a puncture was made. Under the use of astringent injections, the abscess contracted rapidly, and on the 16th of April there remained only a fistulous opening on the outside, and another on the inside, through which a few drops of serum could be pressed, At the end of twelve weeks he was allowed to move the limb in bed, and in four months he walked on crutches, the union being complete. At the present time, August 1st, he moves the member with great facility; the muscles atrophied from long inaction and pressure, are being developed by use; and, although, considerably shortened, it is far superior to any artificial limb.

It will be obvious that the use of the wire in this case, was the only means of avoiding a resort to that extreme resource of art, amputation.

This case is offered to the consideration of the profession, as indicating an additional curative means in a class of cases which sometimes resist the skilful application of every plan heretofore made use of.

